# Student Leadership in Undergraduate Nursing Research: A Qualitative Content Analysis of Roles, Challenges and Professional Growth

**DOI:** 10.1002/nop2.70712

**Published:** 2026-08-06

**Authors:** Allan M. Manaloto, Rhenelee C. Viray, Ramon D. Laderas, Alex S. Borromeo

**Affiliations:** ^1^ College of Nursing, Bulacan State University City of Malolos Bulacan Philippines

**Keywords:** leadership development, nursing professional development, qualitative content analysis, research mentorship, undergraduate nursing education

## Abstract

**Aim:**

To explore the roles, challenges and perceived professional growth of undergraduate nursing student research leaders during thesis work.

**Design:**

This study used a qualitative descriptive design.

**Methods:**

Written reflections from 29 undergraduate nursing student research leaders were analysed using inductive qualitative content analysis. Participants had served as designated research group leaders during undergraduate thesis work. Data were coded, grouped into categories and synthesized into themes. Strategies to support analytic rigour included reflexive journaling, peer debriefing and an audit trail.

**Results:**

Eight themes were identified: roles and responsibilities of group leaders, emotional landscape of leadership, academic and research‐related challenges, group dynamics and interpersonal processes, leadership development and evolving leadership styles, faculty mentorship and institutional support, personal and professional growth, and lessons and recommendations for the future. Findings suggest that undergraduate thesis leadership may strengthen communication, accountability, resilience, collaboration and readiness for future professional roles.

**No Patient or Public Contribution:**

There was no patient or public contribution in the design, conduct, analysis or reporting of this study.

## 1 Introduction

Undergraduate nursing thesis work is increasingly recognized as more than an academic requirement; it can also foster research competence, evidence‐based thinking and early leadership development (Grønning et al. 2022). Students who serve as research group leaders often coordinate peers, communicate with advisers and manage timelines while navigating topic development, revision and data collection (Grønning et al. 2022; Wu and Cormican 2021; Tuppal et al. 2022). These responsibilities may strengthen collaboration, accountability and resilience, yet they may also expose students to stress, self‐doubt and interpersonal strain (Abdul‐Rahim et al. 2025; Labrague 2024).

Although previous studies have examined thesis experiences, supervision and resilience in nursing students (Aryuwat et al. 2024; Jaensson et al. 2024), these have largely centred on students as supervisees or on the supervisor–student relationship. Far less is known about the students who take on the designated leadership role within research groups—those who coordinate peers, manage deadlines and mediate between members and advisers while remaining peers themselves. In the Philippine context, where outcomes‐based curricula mandated by the Commission on Higher Education commonly rely on group‐based thesis models, this dual peer‐and‐leader position is widespread yet rarely documented, particularly with respect to how leaders reflect on coordination, supervisory relationships, group conflict and their own growth after completing the thesis. This study addressed that gap by exploring the reflections of nursing student research group leaders during undergraduate thesis work and considering how these insights may inform mentorship, instructional support and early leadership preparation in undergraduate nursing education and nursing professional development. This study was guided by the following research question: How do undergraduate nursing student research group leaders describe their roles, challenges and perceived professional growth during thesis work?

## 2 Methods

### 2.1 Research Design

This study used a qualitative descriptive design to examine student leaders' written reflections on their undergraduate thesis experiences. Qualitative description is well suited to research that aims to provide a straightforward, comprehensive account of a phenomenon in the everyday language of those who experienced it, without the theoretical commitments of phenomenology, grounded theory or ethnography (Doyle et al. 2020; Sandelowski 2000). Within this design, inductive qualitative content analysis following Elo and Kyngäs (2008) was applied as the analytic method, allowing categories and themes to emerge directly from participants' written reflections rather than from a predetermined framework. This analytic approach was further informed by contemporary guidance on inductive content analysis, which emphasizes systematic familiarization with the data, transparent coding, iterative category development and clear documentation of analytic decisions to strengthen rigour and auditability (Vears and Gillam 2022).

### 2.2 Participants and Setting

The study was conducted in a Bachelor of Science in Nursing programme at a higher education institution in the Philippines. Participants were incoming fourth‐year nursing students who had completed and defended their undergraduate theses during the previous academic level and had served as designated research group leaders. Because the eligible population was small and clearly defined, a census (total enumeration) approach was used, in which all eligible individuals were invited rather than a sample drawn from them; this ensured comprehensive coverage of the entire eligible group. Eligible leaders were identified from a class list provided by the program coordinator and were invited to participate directly by the researcher. Participation was voluntary, and the researchers held no supervisory or evaluative authority over the participants in relation to the data collected. All 29 eligible student leaders agreed to participate.

Eligible participants were incoming fourth‐year (Level IV) BSN students who had served as the designated research group leader during their undergraduate thesis and whose thesis had been successfully completed and defended within the preceding academic year. Students who had not served as designated group leaders, or whose thesis had not been completed and defended within that period, were excluded.

In the program studied, designated group leaders coordinated their thesis teams across the full research process—from topic development through manuscript revision and data collection—serving as the primary liaison between members and the faculty adviser and carrying accountability for group progress. Faculty supervision was available throughout but varied in consistency across groups. Research advisers awarded additional academic points to the designated leader in recognition of this added responsibility; no other form of recognition was attached to the role. This academic credit related to performance of the leadership role during the thesis and was independent of participation in the present study, which was voluntary and carried no incentive.

### 2.3 Data Collection

Data were gathered using a researcher‐developed self‐administered online questionnaire with open‐ended questions comprising 13 items organized into six domains. The opening section asked participants to describe their leadership role and their initial feelings on assuming it. The second explored academic challenges encountered during the thesis, including topic selection, writing, data gathering and analysis. The third examined group dynamics, covering conflict management, motivation and the evolution of each leader's leadership style. The fourth addressed faculty mentorship and institutional support, including advising, deadlines, resources and policies. The fifth invited personal reflections on growth, self‐discovered skills and advice for future leaders. The final section gathered recommendations for improving how nursing research is taught and supported, followed by a closing item allowing participants to share anything not covered by the preceding questions. The complete 13‐item self‐administered open‐ended questionnaire is provided as File S1. The instrument was reviewed by two nursing faculty with qualitative research expertise and pilot‐tested with two former research leaders not included in the study. The questionnaire was administered through Google Forms over a four‐week period during the first semester of academic year 2025–2026. Participants completed the form at their own pace, and contact during this period took place solely through the online platform.

### 2.4 Data Analysis

Responses were analysed using Elo and Kyngäs' (2008) framework for inductive qualitative content analysis, supplemented by recent methodological guidance on inductive content analysis (Vears and Gillam 2022). The analysis proceeded in three phases: preparation, organization and reporting. In the preparation phase, the research team read the reflections repeatedly to become familiar with the data and identified meaning units. In the organization phase, open coding was carried out, and codes were grouped into subcategories and then into broader themes through iterative comparison. In the reporting phase, themes were presented alongside representative participant excerpts. Analysis was conducted collaboratively by the research team; coding decisions and the developing category structure were discussed among the authors, and any disagreements were resolved through discussion until consensus was reached. Analytic rigour was supported using the criteria of Lincoln and Guba (1985) and contemporary recommendations for transparent inductive content analysis (Vears and Gillam 2022). Credibility was addressed through peer debriefing among the research team, repeated engagement with the written reflections, and the use of representative verbatim excerpts. Dependability and confirmability were supported through an audit trail and reflexive journaling that documented coding decisions, category development and analytic discussions. Transferability was supported through description of the study context and participants. Coding and category development continued iteratively until no new categories emerged and the thematic structure was considered stable.

## 3 Results

Analysis of written reflections from 29 nursing student research leaders yielded eight themes describing leadership as academically demanding, emotionally intense and professionally formative.

### 3.1 Theme 1: Roles and Responsibilities of Group Leaders

Participants described the role as multifaceted, involving task coordination, role assignment, adviser communication and decision‐making. Leadership extended beyond delegation to monitoring progress and carrying accountability for group outcomes. As one participant noted, ‘I acted as the bridge between our group and our adviser’ (L5). Another echoed this, recalling, ‘Since high school, I've really enjoyed being the bridge between our teachers and classmates, and I applied the same role here’ (L18).

### 3.2 Theme 2: Emotional Landscape of Leadership

Leadership was described as emotionally complex. Many participants initially reported fear, pressure, and self‐doubt, but later described pride, motivation, and increased confidence as they adapted to the role. One participant shared, ‘I was scared because I didn't think I could handle the pressure’ (L2). Over time, this gave way to pride, as another reflected, ‘I was proud when my group entrusted me to lead’ (L10).

### 3.3 Theme 3: Academic and Research‐Related Challenges

Participants identified topic selection, repeated revisions, data gathering, and statistical analysis as major academic challenges. Several described the process as technically demanding and stressful, especially when group members held differing views or external support was difficult to access. As reflected by one participant, ‘Honestly, every chapter was a challenge. You really need to revise again and again’ (L3). Another described the same strain: ‘For me, one of the most challenging parts of our thesis was the continuous revisions’ (L14).

### 3.4 Theme 4: Group Dynamics and Interpersonal Processes

Student leaders managed conflict, uneven participation, and low initiative while trying to preserve cooperation and fairness. Common strategies included open communication, encouragement, and equitable task distribution. One participant explained, ‘Of course, there were conflicts, but we tried to settle them by talking as a group’ (L6). The source of friction was often relational, as another noted: ‘There were misunderstandings and personal issues that sometimes affected our progress’ (L2).

### 3.5 Theme 5: Leadership Development and Evolving Leadership Styles

Many participants described a shift from simple task delegation to more adaptive leadership approaches that balanced collaboration with firmness when deadlines and accountability required it. One participant recalled, ‘At first, I was hands‐off, but later I became more active’ (L4), while another described moving toward a participatory style: ‘My leadership style evolved into one that was democratic’ (L5). Several reported becoming more active, structured, and intentional in how they led their groups over time.

### 3.6 Theme 6: Faculty Mentorship and Institutional Support

Faculty guidance emerged as either a major source of support or, when less available, an additional source of stress. Institutional deadlines, resource limitations, and policy‐related barriers also shaped the research experience. One participant noted, ‘Our adviser is very supportive and hands‐on, especially when we struggled’ (L5). Others experienced the opposite, with one observing, ‘Sometimes it felt like there was no support at all’ (L1).

### 3.7 Theme 7: Personal and Professional Growth

Despite the challenges, participants described increased patience, confidence, resilience, communication, and problem‐solving. One participant reflected, ‘I developed stronger communication and problem‐solving skills’ (L21). For another, growth meant uncovering unrecognized strengths: ‘I realized hidden skills in myself’ (L6).

### 3.8 Theme 8: Lessons and Recommendations for the Future

Participants advised future student leaders to stay organized, manage time well, and trust their ability to lead. They also recommended clearer research guidelines, stronger preparation in methodology and statistics, and more regular faculty consultation schedules. As one participant emphasized, ‘My advice is to manage your time well’ (L3). Another offered practical guidance: ‘Be organized, keep clear timelines. Communicate well with the group’ (L19).

## 4 Discussion

### 4.1 Roles, Emotions and Leadership Evolution

Student leaders characterized their role as multi‐dimensional project management, serving as liaisons between groups and advisers while bearing accountability for final decisions. This boundary‐spanning responsibility reflects what Ädel et al. (2023) described in thesis supervision, where roles often overlap. Leaders' reflections suggest they carried out scaffolding roles among peers (Karlsholm et al. 2024). Leadership legitimacy emerged through faculty appointment and peer election, combining institutional authority with peer trust (Wu and Cormican 2021).

The emotional landscape was marked by initial fear and anxiety alongside eventual confidence and pride. This progression mirrors broader patterns: nursing students experience moderate to high anxiety in academic leadership roles, yet emotional intelligence transforms stress into growth opportunities (Zheng et al. 2022; Shubayr and Dailah 2025). The developmental journey from reluctance to confidence is consistent with findings that anxiety decreases as competence increases and resilience strengthens with experience (García‐Velasco et al. 2025; Mayor‐Silva et al. 2024).

Leadership styles evolved from tentative task delegation to adaptive approaches blending collaborative and directive strategies. This reflects adaptive leadership, where leaders switch between assertive decision‐making and collaboration depending on circumstances (McKimm et al. 2023). Such flexibility aligns with situational leadership theory (Wang et al. 2024).

### 4.2 Academic Challenges and Resource Constraints

Leaders faced persistent academic obstacles including topic selection difficulties, manuscript revisions, data collection barriers and statistical analysis struggles. These reflect the cognitive and technical demands of undergraduate research. Nursing students often have positive attitudes toward evidence‐based practice yet lack statistical appraisal skills (Li et al. 2024; Lytsy et al. 2022). Continuous revisions and limited resources increased stress (Hwang et al. 2022), while participant refusal and logistical challenges caused data collection delays.

Resource limitations were particularly pronounced. Leaders lacked updated resources and scholarly database access—challenges consistent with structural barriers in low‐ and middle‐income countries including reduced infrastructure and outdated materials (Zhang et al. 2023). Embedding librarians into research courses can help navigate these gaps (Pleshkan and Singarella 2022).

### 4.3 Group Dynamics and Interpersonal Management

Leaders managed complex group relationships, mediating conflicts, addressing passivity and nurturing cooperation. These interpersonal challenges required conflict resolution skills—a vital yet underdeveloped competency in nursing leadership education (Abdul‐Rahim et al. 2025). Low participation reflected weak accountability (Luo et al. 2021), yet leaders employed motivational strategies including goal reminders and fair task distribution. Evidence shows cooperative learning environments strengthen motivation and performance (Lee et al. 2025; Eskiyurt and Özkan 2024).

### 4.4 Faculty Mentorship and Institutional Support

Experiences with faculty guidance varied considerably. Supportive advisers fostered focus and perseverance (Tuppal et al. 2022; Karlsholm et al. 2024). However, supervision gaps created frustration—a pattern consistent with findings that nursing students enter thesis work with high expectations often unmet (Jaensson et al. 2024). Institutional deadlines provided structure while elevating stress (Labrague 2024; Hwang et al. 2022).

### 4.5 Personal and Professional Growth

Despite challenges, leaders gained patience, confidence, resilience and self‐awareness—transforming struggles into professional competencies. This developmental progression mirrors findings that challenging environments cultivate nursing students' relational and empathic skills (Mayor‐Silva et al. 2024). These findings suggest that early academic leadership experiences may contribute to readiness for professional roles by strengthening communication, accountability, adaptability and confidence.

### 4.6 Limitations

This study was limited to a single higher education institution in the Philippines, which may restrict transferability to other nursing programs. Written reflections collected through an online questionnaire did not allow follow‐up probing. The study focused exclusively on student leaders' perspectives without triangulation from group members or faculty advisers.

### 4.7 Implications for Nursing Professional Development Educators

These findings have practical implications for nursing professional development educators and undergraduate nursing faculty. The competencies student leaders described developing—communication, delegation, accountability, conflict management and resilience under pressure—are the same relational and coordinating capacities required of nurses who lead care teams, coordinate across disciplines and maintain safe practice in clinical settings (Jack et al. 2022; Abdul‐Rahim et al. 2025). Framed this way, undergraduate research leadership is not only an academic exercise but an early, low‐stakes rehearsal of clinical leadership, offering a practical avenue for cultivating leadership readiness before students enter practice. Educators may strengthen these outcomes by implementing pre‐thesis leadership orientation workshops, structured research guidebooks, regular faculty consultation schedules, writing and data‐analysis support and stress‐management strategies. Embedding peer mentoring and reflective debriefing into the thesis process may further help students consolidate these experiences into transferable leadership competencies for future nursing roles.

## 5 Conclusion

This study examined the reflections of nursing students who served as research group leaders during their undergraduate thesis. Eight themes emerged, revealing that leadership encompassed task coordination, emotional navigation, academic problem‐solving, interpersonal mediation, faculty relationship management and professional growth. These findings illuminate the dual role student leaders occupy—as both peers and facilitators—and highlight the institutional and pedagogical factors that shape how they are prepared for future leadership and the transition to practice. Despite initial anxiety, student leaders developed confidence, resilience, patience and adaptability—competencies relevant to leadership development and nursing practice.

Success depends on structured preparation, consistent faculty mentorship, adequate institutional resources and recognition of the emotional and interpersonal dimensions of leadership. By integrating leadership orientation, regular consultation schedules, research guidebooks and stress‐management support, nursing programs may strengthen both research learning and professional growth. These findings suggest that undergraduate research leadership can serve as a formative experience that helps prepare future nurses for collaborative practice, accountability and resilience.

## 6 Recommendations

Building on these findings, several recommendations are offered. For nursing education and practice, programs should provide structured leadership preparation before thesis work, including orientation on coordination, conflict resolution and accountability, so that students enter the role with foundational competencies rather than acquiring them solely through trial and error. At the institutional level, schools should ensure consistent faculty mentorship, accessible consultation schedules and adequate research resources, including updated references and statistical support, to reduce the structural barriers that participants identified. For future research, longitudinal and multi‐institutional studies are needed to trace how leadership develops over time and how it varies across settings with differing resource levels; studies incorporating the perspectives of group members and faculty advisers would allow triangulation beyond the leader's viewpoint; and intervention studies could test whether structured leadership‐preparation programs improve research outcomes and practice readiness.

## Author Contributions

Allan M. Manaloto: conceptualization, methodology, formal analysis, investigation, writing – original draft, writing – review and editing, supervision, project administration. Alex S. Borromeo: methodology, formal analysis, validation, writing – review and editing. Rhenelee C. Viray: formal analysis, investigation, data curation, writing – review and editing. Ramon D. Laderas Jr.: formal analysis, investigation, data curation, writing – review and editing. All authors read and approved the final manuscript.

## Funding

The authors have nothing to report.

## Ethics Statement

This study was reviewed and approved by the Bulacan State University Ethics Review Committee (BulSU‐ERC; protocol code 788‐qs8‐u). Ethics clearance was obtained before data collection. Participation was voluntary, and electronic informed consent was obtained from all participants through the opening page of the Google Form before participation. Participants were informed of the study purpose, procedures, potential risks and benefits and their right to decline or withdraw without consequence. No identifying information was collected. Responses were de‐identified, stored securely, and will be deleted 1 year after study completion. The study posed minimal risk, and participants were allowed to skip any question that caused discomfort.

## Conflicts of Interest

The authors declare no conflicts of interest.

## Supporting information


**File S1:** Self‐administered open‐ended questionnaire.

## Data Availability

The data that support the findings of this study are not publicly available due to privacy and confidentiality considerations but may be made available from the corresponding author upon reasonable request and subject to institutional approval.
