# Supplementary material for: Student Leadership in Undergraduate Nursing Research: A Qualitative Content Analysis of Roles, Challenges and Professional Growth
Source: Nurs Open. 2026 Aug 6;13(8):e70712. doi: 10.1002/nop2.70712 (PMC13448138; doi:10.1002/nop2.70712)
Supplement: Supplementary file 1 [file NOP2-13-e70712-s001.docx]

**File S1**

**Self-Administered Open-Ended Questionnaire**

*Manuscript title: Student Leadership in Undergraduate Nursing Research: A Qualitative Content Analysis of Roles, Challenges, and Professional Growth*

**Goal of the Study**

The main objective of this study is to explore and describe the lived experiences and reflections of nursing research group leaders following the completion of their undergraduate thesis. It aims to identify the academic, interpersonal, and structural challenges they encountered, the support systems they utilized or needed, and the growth opportunities they perceived throughout the thesis process.

**Respondent Criteria**

The study was conducted in a higher education institution in the Philippines, focusing on a Bachelor of Science in Nursing program. Inclusion criteria require that participants must have served as a group leader during the conduct of their undergraduate thesis and must have completed the thesis within the most recent academic year. Group leaders should have actively assumed responsibilities such as coordinating tasks, facilitating communication, and managing research timelines. Participants will be selected regardless of sex, academic honors, or group size. Those who did not take on leadership roles or who have not completed their thesis will be excluded to ensure that the data reflects the authentic experiences of those who directly managed the group research process.

**Participant Introduction**

Thank you for completing this questionnaire. The purpose of this study is to understand your personal experiences and reflections as a group leader during your undergraduate nursing thesis journey. There are no right or wrong answers—please feel free to share your honest thoughts and insights. Your responses will be kept confidential and used solely for research purposes.

**Section 1: Opening and Contextual Questions**

1. Can you briefly describe your role as a group leader during your thesis journey?

*Follow-up prompt:: How was the leadership role assigned or assumed?*

2. How did you feel when you first started the research course and learned about the responsibilities of being a group leader?

**Section 2: Academic Experiences and Challenges**

3. What were the most significant academic challenges you and your group encountered while working on your thesis?

*Follow-up prompt:: Were these challenges related to topic selection, writing, data gathering, or analysis?*

4. How did your experience as a leader influence your understanding of the nursing research process?

*Follow-up prompt:: In what ways did the experience enhance or challenge your grasp of research concepts?*

**Section 3: Interpersonal and Group Dynamics**

5. Can you describe the group dynamics throughout the thesis process?

*Follow-up prompt:: How did you manage conflicts, motivation, or workload distribution?*

6. How did your leadership style evolve while working with your group?

*Follow-up prompt:: What strategies worked in keeping the group focused and cooperative?*

**Section 4: Faculty Mentorship and Institutional Support**

7. How would you describe the support and guidance provided by your research adviser or faculty during the thesis process?

*Follow-up prompt:: What worked well? What could have been improved?*

8. What institutional or administrative factors (e.g., deadlines, resources, policies) helped or hindered your group’s progress?

*Follow-up prompt:: Were there any moments where you felt lost or unsupported?*

**Section 5: Personal Reflections and Growth**

9. Looking back, what did you learn about yourself as a student and as a leader throughout this journey?

*Follow-up prompt:: Any personal qualities or skills that you discovered or strengthened?*

10. If you were to give advice to future research group leaders, what would it be?

*Follow-up prompt:: What should they expect, and how can they prepare?*

**Section 6: Recommendations for Improvement**

11. Based on your experience, what improvements would you suggest for how nursing research is taught and supported in your program?

*Follow-up prompt:: Consider the roles of instructors, the structure of the course, or the availability of resources.*

12. How can the administration and faculty better support student leaders and thesis groups moving forward?

**Closing Question**

13. Is there anything else you’d like to share about your experience as a research group leader that was not covered in the previous questions?
